# Selection of Mice for Object Permanence Cognitive Task Solution

**DOI:** 10.3390/neurolint14030058

**Published:** 2022-08-29

**Authors:** Olga Viktorovna Perepelkina, Inga Igorevna Poletaeva

**Affiliations:** Biology Department, Moscow State University, Vorobieby Gori, 1, Build. 12, 119234 Moscow, Russia

**Keywords:** cognitive task, genetic differences, puzzle-box, novelty, object permanence, selection

## Abstract

The selection of mice for high (“plus”) and low (“minus”) scores in the puzzle-box test was performed over five generations. This test evaluates the success (or failure) in finding the underpass, leading to the dark part of the box, when it is blocked. This means that the mouse is either able or unable to operate the “object permanence rule” (one of the index’s cognitive abilities). For the “+” strain, animals were bred who solved the test when the underpass test blocked with a plug; the “−” strain comprised those who were unable to solve this task. In mice of the “+” strain, the proportion of animals that was able to solve “plug” stages of the test was higher than in the “−” strain and in the non-selected genetically heterogeneous population. The “+” mice ate significantly more new food in the hyponeophagia test. Animals of both strains demonstrated the ability to “manipulate” the plug blocking the underpass, touching the plug with their paws and muzzle, although the majority of “−” mice were unable to open the underpass effectively. Thus, mice of both selected strains demonstrated that they were able to understand that the underpass does exist, but only “+”-strain animals (at least the majority of them) were able to realize the solution. The selection for plug-stage solution success affected the mouse’s ability to open the hidden underpass.

## 1. Introduction

As stated in one behavior genetics historical article, “validation of behavioral constructs and the ways to test them is urgently needed in both animal and human behavioral and psychiatric genetics” [1]. The term “cognitive” behavior, or the ability to solve a test which requires an adaptive response, is usually addressed to behavioral reactions which require classical and/or instrumental conditioning (sometimes of a very complicated structure). Genetic studies of learning abilities have been performed for many years using rodent selection in food-reinforced learning and aversive learning paradigms. Famous rat selection experiments include those by Tryon [2] and Korochkin et al. [3,4] involving rat selection for instrumental learning in the Novosibirsk Institute for Genetics and Selection. Selection experiments for and against successful aversive learning were also performed in Roman, Syracuse and Hatano strains of rat [5,6,7,8]. Experimental evidence concerning genetic differences in mouse learning performance is widespread (among others [9,10,11]), especially in hippocampus-dependent spatial orientation and memory tests. These results describe interstrain differences in mouse learning using different strains, as well as differences in animals with genetically engineered genotypes. The specific properties of respective neuronal networks were also indicated [12,13,14,15,16,17,18].

The term “cognitive behavior”, used here, refers to the animal’s ability to solve an elementary logic task which is presented to the subject for the first time, i.e., when the subject has no analogous previous experience, as in case of any learning paradigm. So it is not the learned response *per se* to certain environmental signals. The solution of such a task requires “understanding” the elementary logic of the used paradigm. According to Krushinsky’s definition [19], the animal’s “cognitive ability” (or elementary reasoning) is the ability to “grasp” the empirical laws which connect objects and events in the external world and to develop further adaptive behavioral reactions using such information.

The present paper describes the results of an experiment in which mice were selected over five generations for high and low scores in the puzzle-box test solution, first applied to mouse experiments by Galsworthy et al. [20]. The puzzle-box experimental design requires an animal to understand that the object (an underpass, which the mouse is eager to use, as it leads into the safe box compartment) is hidden, being masked by wood shavings or a plug, but can be discovered [21]. The ability to solve the puzzle-box test requires that the animal apply the rule of “object permanence” (according to Piajet, see [22]). The animals in the initial population for this selection experiment were mice of the EX strain. This strain was selected earlier for high scores in the extrapolation task [23]. The correct extrapolation task solution is also based (at least partly) on an animal’s ability to understand the rule of “object permanence”. In the mental operation of “extrapolation”, the animal has to find the new location of food bait on the basis of information perceived, although the food bait is no longer seen, as it has been moved away from view to the right or to the left of the animal, which perceives the food bait via a small opening at the base of the box’s front wall.

In order to make the selection routine more feasible, we simplified the puzzle-box procedure so that the experimental testing could be performed over one day, in comparison to the longer test schedules used initially [20,21].

The selection criterion for the “plus” strain was short latency of test solutions at test stages when the underpass was masked by a plug, and the lack of these solutions (in an arbitrary time interval of 240 s) in the “plug” stages was the criterion for the strain selected as “minus”. Previously published data [24] demonstrated success in this selection process for F1–F3. The behavior of mice from “plus” and “minus” strains was also compared in a hyponeophagia test [25]; the amount of new food eaten during the test time was persistently higher in “plus” mice. In the hyponeophagia test, the new food (small cubes of cheese) was presented to the hungry mouse in a new, but not frightening, environment. Animal behavior in this test is affected by both the necessity of handling the “novelty” (new food) and the “concurrent” anxiety reaction, which is aroused by the new environment (and the novelty of cheese as a food) and could obscure the reaction to this novelty. This test has been successfully used to evaluate the effects of antidepressants [26], and was earlier regarded as a test for anxiety [27].

Thus, the results of the five-generation selection for this cognitive trait in mice are presented below. The working memory indices, which could be drawn from the protocol of the puzzle-box solution, were also analyzed.

## 2. Material and Methods

Experimental animals. Mice were bred selectively starting from F20 of the strain, previously selected for high scores in the extrapolation test (see above). All animals, born in each generation (males and females), were tested with a simplified puzzle-box test (for details, see below). Animal numbers (for “+” and “−” strains, respectively) were as follows: F1 (“+”) 31 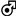

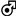
, 29 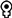

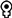
, F1 (“−”) 22 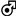

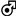
, 17 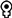

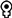
, F2 (“+”), 28 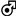

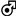
, 30 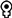

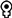
, F2 (“−”) 22 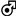

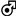
, 27 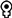

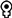
, F3 (“+”) 41 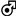

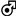
, 42 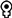

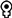
, F3 (“−”) 28 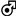

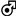
, 18 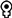

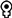
, F4 (“+”), 39 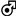

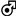
, 33 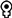

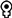
, F4 (“−”) 26 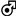

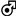
, 18 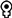

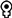
, F5 (“+”) 27 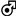

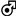
, 39 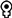

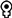
, F5 (“−”) 44 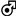

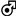
, 47 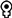

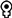
. Some animals in these animal groups from each generation were tested in the hyponeophagia test (the limited number of animals in this test was due to technical problems). Mice in the control, non-selected, heterogeneous populations from the respective generations were also tested in parallel with F4 and F5 of the selected strains (*n* = 97, 63 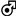

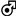
, 34 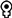

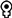
, in total), though F1–F3 control animals were not tested due to a technical problem.

Mice were housed in plastic cages (size 35 × 56 × 20 cm) with food (Firm Laboratorkorm) and water ad libitum with a natural light–dark schedule.

Statement on the welfare of animals. The experimental protocol was accepted by the Bioethical Commission of Moscow State University, session no. 49 of 18 June 2014.

**Figure 1:**
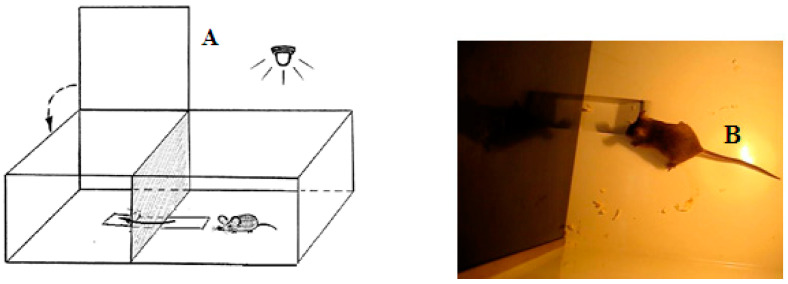
(**A**) Schematic drawing of the puzzle-box experimental box. The brightly lit part of the box (size 30 × 28 × 27.5 cm) was connected with the dark compartment (size 14 × 28 × 27.5 cm) via the underpass (1.5 cm deep, 4.5 cm wide and 11.5 cm long). The arrow indicates the trajectory of an animal while it penetrates the dark part of the box, and another arrow indicates the lid which covers that compartment, providing the comfortable lack of illumination. (**B**) The photo image of the unobstructed, open underpass and a mouse which is ready to enter the dark compartment.

Puzzle-box test. An animal was placed into the brightly lit part of the experimental box, from which it could easily go into the dark part of the box, avoiding the light (see Figure 1). The underpass leading to the dark part of the box was submerged below the floor level and the animal could use it to easily penetrate into a more comfortable, dark compartment.

During test stage 1 of the test, this underpass stayed unobstructed, and the animal could freely enter the dark. At test stage 2, the underpass was masked (covered up to the floor level by fresh wood shavings). At stages 3 and 4, the underpass was blocked by means of a plug (made from carton and plastic), which animals could easily remove by using their teeth, or move aside using a muzzle and paws. The animal was given 180 s to solve stages 1 and 2, whereas for stages 3 and 4 (which required more effort), it was given 240 s. After animal entered the dark part of the box, it was left there for 15–20 s, and then placed in a separate clean cage for 45–60 s before the next stage of the test initiated. The latencies of animal reaction (when entering into the dark part of the box) were registered manually. At stages 3 and 4 (i.e., stages with the plug), the animal movements to remove the plug (“manipulations”, i.e., the attempts to enter the dark by seizing the plug with their teeth, as well as the attempts to raise it) were also registered. In cases when the animal failed to solve the plug stage (for 240 s), the presence or absence of this type of manipulation was considered to be an important index for evaluation of the interstrain behavioral differences. The proportions of mice from the given group which were able to solve stages 3 and 4 of the puzzle-box test were registered as well.

Hyponeophagia test. Animals were food (but not water)-deprived for 10–12 h. The mouse was placed in the dimly lit round arena (diam. 40 cm) surrounded by a plastic wall (height 35 cm), and a small cup with pieces of cheese was placed in the center of this arena. During 5 min of testing, the latency of the first approach to the cheese and the number of approaches and retreats were manually registered, and the amount of cheese consumed during the test was determined.

Selection. After the completion of behavioral testing, “plus” and “minus” groups of male and female mice were chosen as parents for the next generation. The candidates for the “plus” strain needed to successfully solve stages 3 and 4 of the test with latencies not longer than 60–90 s. As practically all mice under study solved test stages 1 and 2 with rather short latencies, the criterion for selection in the “minus” strain was the lack of solutions of both the 3rd and 4th test stages. As the time to penetrate the dark part of the box during stages 1 and 2 was short in all animal groups, these data were not used as selection criteria, although the latencies in selected “plus” animals were shorter.

The future parents of the next generation (1 male and 1 or 2 females) were placed for mating in the cages of a smaller size (39 × 20.5 × 9 cm). The pregnant females were placed in the separate cages and stayed with their litters up to weaning at the age of 30–34 days. Pups were ear-marked and put into the larger cages (with approximately 6–8 animals per cage, males and females separately). The behavioral testing started not earlier than at 3–3.5 months of age, with the puzzle-box test being the first one, followed (for some animals) by the hyponeophagia test.

Statistic differences evaluation. The statistical significance of differences in latency of puzzle-box solutions and of hyponeophagia test scores was evaluated by means of 1- and 2-factorial ANOVA (factors—strain and sex) with the post hoc Fisher LSD test. The differences in proportions of animals which solved the 3rd and 4th stages of the puzzle-box test were evaluated using the Fisher φ-test for alternative proportions difference.

## 3. Results

**Figure 2:**
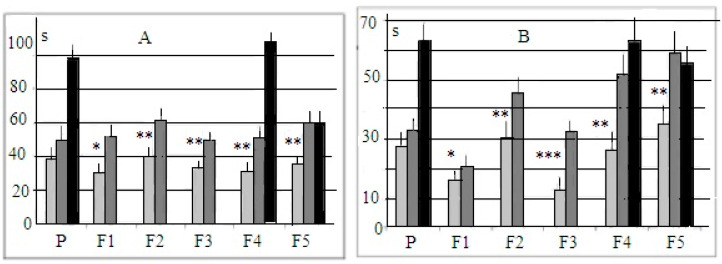
Mean latencies (±st.error, sec, ordinate) for mice entering into the dark part of the experimental box for different groups. (**A**) The underpass was unobstructed. (**B**) The underpass was masked by the wood shavings. Horizontal axis—groups of animals. P—animals of parental generation, i.e., summed performance of mice, chosen for further “plus” and “minus” selection as parents. F1–F5—selection generations. Light gray columns—mice of “plus” selection strain, dark gray columns—mice of “minus” selection strain. Black columns—mice of the control, non-selected, heterogeneous population. *, **, ***—significantly different from the respective values for “minus” and control mice, *p* < 0.05, 0.01 and 0.001 respectively (post hoc Fisher LSD test, one-way ANOVA).

Puzzle-box test. The mean latencies of the puzzle-box test for stages 1 and 2 are presented in Figure 2. The histograms demonstrate that the mean time of entrance into the dark at these (more “simple”) stages of the test were shorter in the “plus” group even in animals of the first selected generation in spite of the fact that parental groups (“P” in Figure 2), i.e., parents for the “+”and “−” selections, solved the test with similar mean latencies. As the puzzle-box stage with the unobstructed underpass is more or less similar to the procedure of the light–dark test, these differences (not large, but statistically significant) reveal that the selection process presumably also affected the expression of anxiety (although this issue was not yet analyzed in detail). During stage 2 of the test (with the underpass masked by the wood shavings), the performance of “plus” mice was quicker than that of “minus” animals.

**Figure 3:**
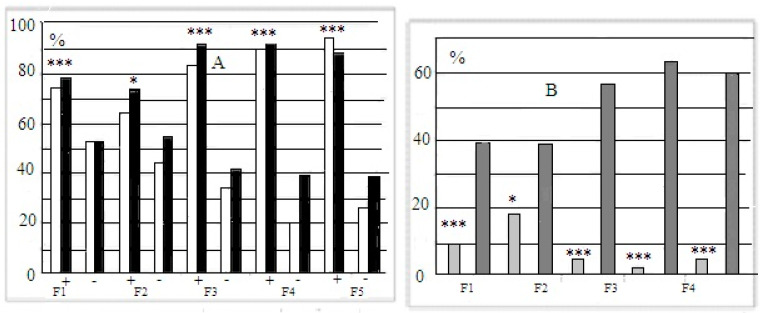
(**A**)—Proportions (%, ordinate) of animals in F1–F5 able to solve the puzzle-boxx when the underpass was blocked by the plug (stages 3 and 4 are designated as white and black columns). (**B**) Summarized data for the proportions (%, ordinate) of animals in F1–F5 that failed to solve both “plug” stages. Designations as in Figure 2. *, ***—significant difference from “minus”-strain scores for both stages, *p* < 0.05 and 0.001, respectively (Fisher φ-method for significance evaluation between alternative proportions).

The mean latencies of “plus” and “minis” mice groups during performance at stages 3 and 4 of this test were significantly different, those of “plus” mice, being shorter (data not presented). The longer latencies of “minus” group mice reflected not only their slower reaction during the test but also the occurrence of cases with 240 s scores for “non-solutions”. Thus, the more adequate evaluation of solution success at the “plug” stages of this test was the proportion of animals of each group that was able to solve the task when the underpass was blocked by a plug. These scores illustrate the resulting interstrain differences better (Figure 3A). Figure 3B presents the summarized proportion scores (for both “plug” stages) for “plus” and “minus” groups that failed to solve the task.

**Figure 4:**
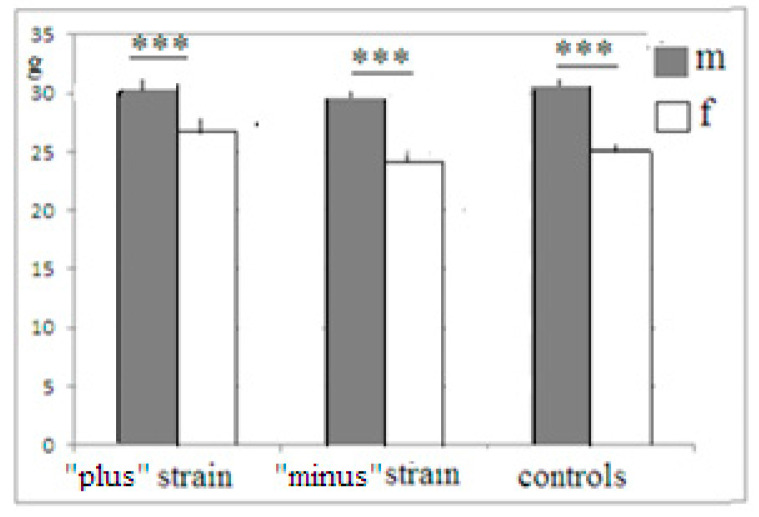
The body weight (ordinate, mean ± stand. err) of male (m) and female (f) mice from F5. ***—significant differences between males and females (one-way ANOVA, LSD Fisher post hoc test).

Mice of both newly selected strains were active in the attempts to manipulate the plug, which blocked the underpass, even those animals which failed to remove the plug successfully. This fact indicates that they all were able to “grasp” the object permanence rule, but animals selected for “plus” solutions were able to bring these attempts to realization. One may suggest that animals of the “minus” group and controls were less adapted for the “plug” stage solution, being smaller and thus less “muscular”. The data for all F5 mice show that this was not the case (Figure 4). In “plus”, “minus” and control groups, males were heavier than females (with a lack of differences in task solution success, data not presented), but the differences in body weight between the same-gender “plus”, “minus” and control mice were absent. Thus, one may conclude that animals of the “plus” groups were actually significantly superior to “minus” mice in the task solution (i.e., incidences of plug removal).

**Figure 5:**
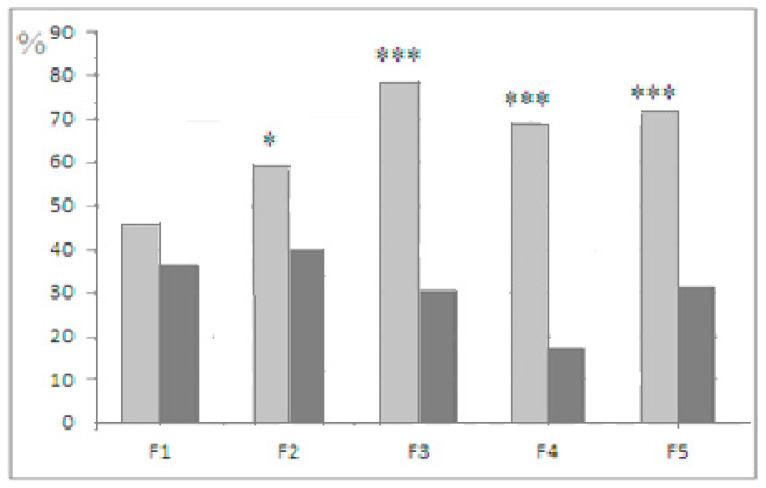
Light gray columns—mice of “plus” selection strain, dark gray columns—mice of “minus” selection strain. Proportions (%, ordinate) of animals in the successive selection generations, which performed stage 4 with shorter latencies than stage 3. *, ***—significant differences between plus and minus strains (Fisher φ-method for significance evaluation between alternative proportions). Designations as in Figure 2.

The comparison of individual solution latencies at stages 3 and 4 (Figure 5) shows that the shortening of stage 4 latencies (from those of stage 3) was more frequent in the “plus” than in the “minus” strain. These data (preliminary, as they only noted the fact of shorter latency, but not the scope of such differences) could not be regarded as a specific test for working memory, but still they demonstrate the interstrain differences in the effect of previous experience—the quicker realization of a solution by “plus” mice during the second “plug” presentation.

The intergenerational comparison is worth mentioning. In F5, the proportion of mice that were able to solve the first “plug” stage (stage 3 of the puzzle-box test) in the “plus” strain was significantly higher than in F1 (t = 2.89, *p* < 0.01, φ test), and the opposite tendency was noted for the respective values in the “minus” strain from F1 to F5, with a decrease in the percentage of animals that solved stage 3 of the test (t = 2.07, *p* < 0.05, φ test).

**Figure 6:**
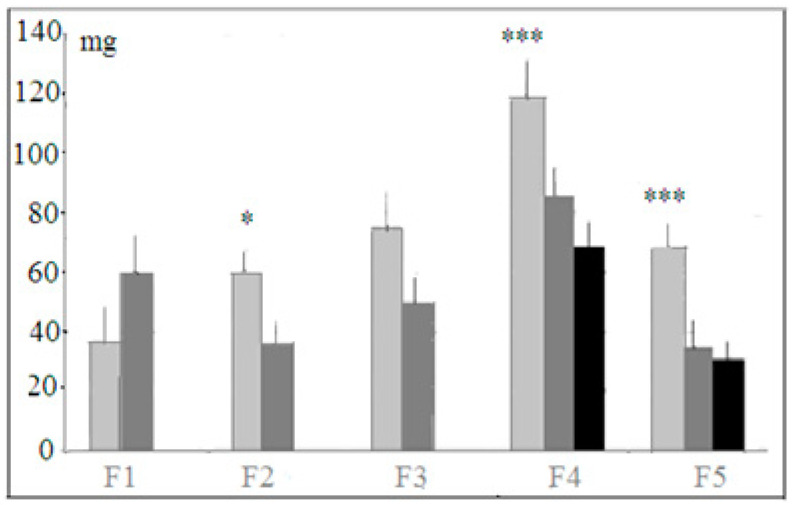
Light gray columns—mice of “plus” selection strain, dark gray columns—mice of “minus” selection strain, black columns—mice of the control, non-selected, heterogeneous population. The amount (ordinate, mgs) of the new food (cheese) eaten by hungry mice in selection generations (horizontal axis) during 5 min of hyponeophagia test. *, ***—significant differences between males and females *p* < 0.05 and 0.001 (one-way ANOVA, LSD Fisher post hoc test) Designations as in Figure 2.

Hyponeophagia test. Among several behavioral indices registered in this test, the amount of food eaten during 5 min of this test revealed the stable differences along selection generations (Figure 6).

Mice of the “plus” strain ate more new food (cheese) than “minus” animals and more cheese than mice of the control non-selected genetically heterogeneous population, although the differences were not statistically significant for F1 and F3. The new food—small cubicles of cheese—was eaten by an animal in the test situation not all at once but during several approaches to the food cup. The numbers of such approaches, as well as time occupied by consuming the new food, varied among generations in non-systemic way (data not presented). The same was true for the number of freezing episodes during this test, the index being lower for “plus” mice. This difference was statistically significant for F5 only (*p* < 0.001, 1.52 ± 0.3 for “plus” strain, 6.7 ± 0.3 for “minus” strain and 5.2 ± 0.7 for mice of the heterogeneous population). These data demonstrate that mice selected for successful solutions of puzzle-box cognitive stages (“plus” strain) were less frightened by the new environment with the new food in comparison to two other groups. These data could be interpreted as showing a positive reaction to novelty, and also higher anxiety in “minus” and control mice (which could be also inferred from longer latencies during stages 1 and 2 of the puzzle-box test; see above).

## 4. Discussion

Data on puzzle-box successes and failures in mice of “plus” and “minus” strains show that definite cognitive ability traits (i.e., solution of “object permanence” task) could be selected for high and low values. The interstrain differences in reactions to new food by a hungry animal in a new environment (hyponeophagia test) could also be an indication of real difference in the cognitive capacity between “plus” and “minus” mice as well. The regular quantitative data have not yet been obtained for cases of plug “manipulations” by “minus” mice (when animals were not able to solve the plug stage), although this type of behavior displayed by “minus” mice in cases of solution failure could be cautiously discussed in the following way. Both “plus” and “minus” mice presumably understand the object permanence rule (that the plug covers and masks the underpass), but “plus” mice are significantly more effective in real solution performance. This could mean that the interstrain differences in plug-stage behavior could be attributed to the differences in executive functions, i.e., the ability (and maybe persistence) to achieve a definite solution. The executive function notion, acquired in psychology, is now applied to animal cognitive behavior as well [21,28,29,30,31,32]. In our case, the executive function behavioral expression was the effective removal of the plug within the arbitrary 240 s time interval.

Data obtained also permit us to suggest that the working memory capacities seem to be affected by this selection. The “plus”-strain populations contained larger proportions of animals that acted more quickly when they were presented with the “plug” stage of the puzzle-box test for the second time than the “minus”-strain mice. These two “plug” presentations were separated from one another by dozens of seconds, and thus, we may attribute these interstrain differences to the differential influence of previous solution success in mice of two selected strains. As was stated by other authors, the working memory could be viewed as a flexible system that both maintains current information and supports the simultaneous execution of higher cognitive functions. The possible variations in the efficacy of working memory [33] could impact individual differences in intelligence scores [34]. Our conclusion concerning working memory differences could be regarded as a preliminary one because it needs confirmation in special experiments (as in, e.g., [35]). Memory genetic differences and mouse reactions to novelty were noted not once in the papers published in the last few decades, e.g., [9,16].

The experimental data obtained during the last few decades provide information concerning the genetic basis of animal behavior, although, apart from data on knock-out and knock-in mice, the problem-solving capacity in genetically different populations has not been analyzed. At the same time, the differences in the ranking of strains in different spatial tasks indicate that no single task can reveal the full richness of spatially guided behavior in a wide range of mouse genotypes [17]. As a whole, the correspondence of scores from different learning tasks in animals of different genotypes is a complicated issue. Animals from strains selected for fear conditioning did not differ in their approaches in conditioning task and in Morris water maze performance, and this probably means that the g-factor [35,36] was not affected by such selection [37], but it may be that the selection affected the motivational basis of such traits. Selection based upon physiological traits, related to the function of the autonomic cholinergic system, was also not accompanied by differences in cognitive function in rats [38]. The hippocampal morphological variability, affecting the mossy fiber synaptic projections in correlation with novelty reactions in Naples selected strains, correlated with processes which modulate strain-characteristic responses to a spatial novelty [39], although these authors indicated that this difference is of a non-genetic origin. At the same time, the genetic correlations of the performance in spatial task with the size of mossy fiber projection was established around the same period of time [40,41]. The QTL technique was also able to identify two loci, on chromosomes 4 and 12, which influenced behavior in a probe trial of the water maze [42]. The extrapolation ability in mice with NCX2 gene knock-out (sodium–calcium exchanger gene 2), tested in our experiments earlier, could demonstrate the participation of cell membrane fine machinery in the expression of such a complicated trait [43]. The relatively simple (i.e., oligo-genic) determination of “learning” genes, inferred from the fact of quick selection response and from differences in genetically defined strains [10,14,44], was not confirmed by further detailed molecular genetic investigations, in which gene expression differences were found to be much more complicated and numerous both in the background state and in situations of spatial learning and fear-based conditioning [44]. Thus, it was not surprising that the attention of neurobiologists shifted to studies in which the role of single genes in complicated behavioral expression of cognitive abilities could be identified. In these works, the improvement in cognitive traits’ expression was detected after gene expression manipulations (among others [45,46]). The participation of definite genetic elements in cognitive traits’ expression was confirmed in many investigations, and attention has now largely shifted to murine models of human diseases in the hope (rather real) of finding therapy approaches [12,18,28,47].

The response to selection in our experiment indicates that there are at least two important points to be aware with. First is the possibility that selection ability in the “plus” strain could vanish in further generations, as was described earlier for selection for mice with high scores on a extrapolation task solution [23], and the second point is (in the case the “plus”–“minus” difference persists) that the elevated executive function in this mouse population was determined by a rather small set of genetic elements. The selection for high and low learning abilities in rodents has been performed several times on the basis of both food and aversive motivations, which is not analyzed here, as it was presented earlier for learning in simpler animals [48]. The differences in g-factor [36,49], especially when the puzzle-box test was used as one of the “units” in a test battery [20,21,50], is also a point of interest. These data being applied to the definite genetic groups of animals may lead to identifying the genetic elements involved in cognitive task solutions [11]. The prefrontal cortex function in the aspect of cognitive ability, working memory and other complicated brain functions is also the focus of investigations with promising results [29,51]. The ability to understand the object permanence rule was recently analyzed in “Comparative cognition in three understudied” ungulate species—European bison, forest buffalos and giraffes [52]. The authors note that such data are important for understanding the evolution of animal cognition. An interesting development of these ideas was recently described by an analysis of trait inheritance in birds, namely pheasants, which are not generally used to study behavior genetics. The heritability and correlations of learning and inhibitory control traits were analyzed in 450 pheasants, *Phasianus colchicus*, over four generations [53], with the data clearly demonstrating the necessity of more broad analysis of this issue.

## Data Availability

We do not analyze or generate any datasets, because our work is experimental and describes the respective results. All data generated or analyzed during this study are included in this published article.
